# High-Load and Low-Load Resistance Exercise in Patients with Coronary Artery Disease: Feasibility and Safety of a Randomized Controlled Clinical Trial

**DOI:** 10.3390/jcm11133567

**Published:** 2022-06-21

**Authors:** Tim Kambic, Nejc Šarabon, Vedran Hadžić, Mitja Lainscak

**Affiliations:** 1 Cardiac Rehabilitation Unit, Department of Research and Education, General Hospital Murska Sobota, Ulica dr. Vrbnjaka 6, Rakičan, 9000 Murska Sobota, Slovenia; 2 Faculty of Health Sciences, University of Primorska, Polje 42, 6310 Izola, Slovenia; nejc.sarabon@fvz.upr.si; 3 InnoRenew CoE, Human Health Department, Livade 6, 6310 Izola, Slovenia; 4 S2P, Science to Practice, Ltd., Laboratory for Motor Control and Motor Behavior, Tehnološki Park 19, 1000 Ljubljana, Slovenia; 5 Faculty of Sport, University of Ljubljana, Gortanova Ulica 22, 1000 Ljubljana, Slovenia; vedran.hadzic@fsp.uni-lj.si; 6 Division of Cardiology, General Hospital Murska Sobota, Ulica dr. Vrbnjaka 6, Rakičan, 9000 Murska Sobota, Slovenia; 7 Faculty of Medicine, University of Ljubljana, Vrazov Trg 2, 1000 Ljubljana, Slovenia

**Keywords:** strength training, aerobic training, cardiac rehabilitation, hemodynamic response, acute coronary syndrome, exercise training

## Abstract

Resistance exercise (RE) remains underused in cardiac rehabilitation; therefore, there is insufficient evidence on safety, feasibility, and hemodynamic adaptations to high-load (HL) and low-load (LL) RE in patients with coronary artery disease (CAD). This study aimed to compare the safety, feasibility of HL-RE and LL-RE when combined with aerobic exercise (AE), and hemodynamic adaptations to HL and LL resistance exercise following the intervention. Seventy-nine patients with CAD were randomized either to HL-RE (70–80% of one-repetition maximum [1-RM]) and AE, LL-RE (35–40% of 1-RM) and AE or solely AE (50–80% of maximal power output) as a standard care, and 59 patients completed this study. We assessed safety and feasibility of HL-RE and LL-RE and we measured 1-RM on leg extension machine and hemodynamic response during HL- and LL-RE at baseline and post-training. The training intervention was safe, well tolerated, and completed without any adverse events. Adherence to RE protocols was excellent (100%). LL-RE was better tolerated than HL-RE, especially from the third to the final mesocycle of this study (Borgs’ 0–10 scale difference: 1–2 points; *p* = 0.001–0.048). Improvement in 1-RM was greater following HL-RE (+31%, *p* < 0.001) and LL-RE (+23%, *p* < 0.001) compared with AE. Participation in HL-RE and LL-RE resulted in a decreased rating of perceived exertion during post-training HL- and LL-RE, but in the absence of post-training hemodynamic adaptations. The implementation of HL-RE or LL-RE combined with AE was safe, well tolerated and can be applied in the early phase of cardiac rehabilitation for patients with stable CAD.

## 1. Introduction

Exercise-based cardiac rehabilitation (CR) presents a cornerstone of secondary prevention for patients with coronary artery disease (CAD) [1], with aerobic exercise (AE) and resistance exercise (RE) recommended as core components [1,2,3]. While AE has been widely implemented and used [4], RE remains underused due to the lack and/or absence of specific guidelines among leading CR associations [4], poorly structured RE interventions in the previous studies [5,6], the absence of reports on safety and adherence [5,6], and due to safety concerns related to the enhanced risk of cardiovascular events [7,8,9]. For illustration, nearly a half of the recommendations published by the leading cardiac rehabilitation organizations have not included RE as an exercise modality in CR and/or have not specified the exact exercise recommendations (e.g., training intensity, frequency, and duration) for RT. In addition, most previous trials have also failed to adequately report adherence to exercise and potential adverse events [4,5,6]. Therefore, the recent developments of AE programs were not followed by the advances in design of RE programs [2,4].

Despite such drawbacks in implementation, RE was shown to be associated with lower mortality [10] and studies have demonstrated the beneficial effects of combined AE and RE on patients’ maximal physical performance, body composition and quality of life when compared with AE as a standard care [5,6]. However, these studies applied only low-load (LL) RE (<40% of one-repetition maximum [1-RM]) to moderate-load RE (40–60% of 1-RM) [5,6], which may present a suboptimal training stimulus compared with high-load (HL) RE (>70% of 1-RM) otherwise advised for healthy older adults [11,12]. HL-RE has shown superior effects on muscle strength compared with LL-RE in healthy young and older adults [13,14], while such effects remain to be investigated in patients with CAD. In addition, limited studies have balanced the training volume between HL-RE and LL-RE to focus solely on the training load in healthy older adults [14].

Traditionally, participation in RE was assumed to be associated with increase cardiovascular risk (e.g., excessive increase in heart rate (HR), blood pressure and cardiac output) [7,15], which was only recently proven not to be the case in patients with CAD [16,17,18,19,20]. In contrast to this common belief, hemodynamic studies have demonstrated that HL-RE (70–90% 1-RM) elicits lower HR, blood pressure and rating of perceived exertion (RPE) compared with low- to moderate-load RE (35–60% 1-RM) [16,17]. Since all previous evidence is based on already trained patients with CAD [16,17,18], we have recently demonstrated that both types of RE are safe and well tolerable in patients with CAD prior to enrolment to CR [19]. Nevertheless, it still remains unknown whether the early implementation of HL-RE in CR provides any favorable hemodynamic adaptations and potentially improves exercise tolerance.

On this basis, this secondary analysis of a randomized controlled, clinical trial [21] consisted of two aims. Firstly, this study aimed to compare the safety and feasibility of HL-RE and LL-RE combined with AE and compared with AE. Secondly, our study aimed to investigate the effects of different exercise training modalities on 1-RM, and hemodynamic adaptations (e.g., HR) and exercise tolerability (e.g., RPE [Borgs’ scale 0–10 points]) during HL-RE and LL-RE following the training intervention. Therefore, we have hypothesized that HL-RE and LL-RE when combined with AE will be safe and feasible. HL-RE was expected to induce greater improvement in 1-RM compared with combined AE and LL-RE or AE alone. In addition, we also expected an improvement in exercise tolerance during post-training HL-RE and LL-RE, with an absence of hemodynamic adaptations to both.

## 2. Materials and Methods

### 2.1. Study Design

**Figure 1:**
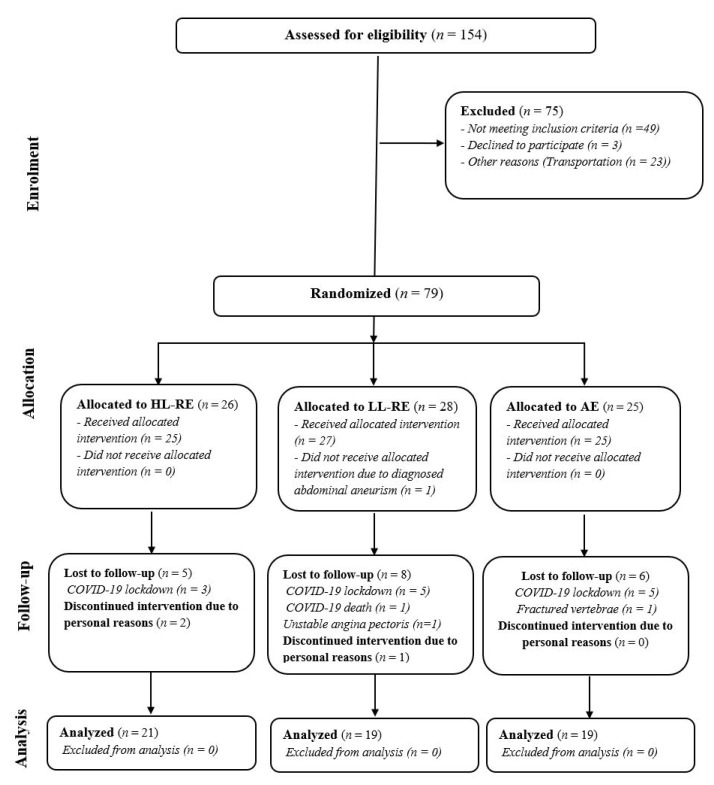
CONSORT study flow. HL-RE: high-load resistance exercise; LL-RE: low-load resistance exercise; AE: aerobic exercise; COVID-19: coronavirus disease-19.

This study was designed as a randomized controlled, clinical trial with three parallel arms (Figure 1): HL-RE combined with aerobic interval exercise; LL-RE combined with aerobic interval training; and aerobic interval training as a standard care. The design of this study was prepared in accordance with the Consolidated Standards of Reporting Trials (CONSORT) guidelines [22] and has been published previously [21]. After baseline clinical assessment, patients were cluster randomized. During this study, the clusters were adjusted from 5:5:5 to 3:3:3 patients for safety reasons associated with the ongoing coronavirus-19 pandemic. 

The outcomes of this secondary analysis were safety and adherence to the training intervention, cumulative workload completed during AE and RT, change in 1-RM on leg press machine after 7 weeks and post-training, and hemodynamic adaptation to LL-RE and HL-RE.

We assessed patients at baseline (during the first three training sessions), after 7 weeks and post-training (during the last three training sessions). Prior to baseline, we have also familiarized patients with proper lifting and breathing technique on leg press machine to avoid potential activation of Valsalva maneuver [7,9,23]. These protocols took place during the measurement days outside the aims of this study and can be accessed elsewhere [21]. In this study, we assessed patients maximal leg press strength (1-RM) and hemodynamic response and RPE during LL-RE and HL-RE at first, second and third training sessions, respectively. Apart from re-evaluation of 1-RM after 7 weeks (on 22nd session), all measurements were repeated during the last three training sessions in a reversed order (hemodynamic response to LL-RE and HL-RE followed by 1-RM evaluation). We permitted patients to be engaged in low- to moderate-intensity physical activity at home during the rest days (walking, cycling, calisthenics, etc.), with the exception of RE.

### 2.2. Participants

Patients with CAD (acute coronary syndrome and/or percutaneous coronary intervention) were recruited from the Division of Cardiology, General Hospital Murska Sobota, Slovenia. Inclusion criteria were age 18–85 years, left ventricular ejection fraction ≥ 40%, documented CAD, time from clinical event (≥1 month), referral to phase II out-patient CR and completion of a baseline cardiopulmonary exercise test [1,24]. Exclusion criteria followed standard recommendations for participation in RE [15,23].

### 2.3. The Training Intervention

Patients underwent three training sessions per week for 12 weeks or a total of 36 training sessions (60–70 min/session), with at least 48 h rest between sessions. Each training session consisted of a general warm-up (10 min dynamic flexibility exercises followed by calisthenics using elastic bands and/or LL dumbbells and balance exercises), AE (35–40 min) and RE (5–10 min) and cool down (5 min static stretching and breathing exercises). In the main part of each session, all patients performed aerobic interval cycling (3–5 min workload cycling separated by 2 min unloaded cycling) starting from the initial 50% of maximal workload achieved at baseline cardiopulmonary exercise test and progressively increasing every two weeks to 80% maximal workload [1,25]. Duration of workload interval during AE decreased from 5 min to 4 min after 6 weeks (on 19th session), and from 4 min to 3 min after 10 weeks (on 30th session). Cycling cadence was set at 50–60 revolutions per min [1,21].

Patients in both the RE groups completed a total of 36 sessions on a leg press machine (three 1-RM tests and 33 RE sessions). The training load differed between the two groups; training volume was balanced by the number of repetitions. The range of number of repetitions was in line with previous recommendations for RE in CR [1,7,23]. Overview of measurements and training protocol is displayed in Table 1.

In brief, we first familiarized patients with RE before baseline testing to ensure their use of correct lifting and breathing techniques, and aiming to avoid the Valsalva manoeuvre [7,9,23]. In the HL-RE group, workload was increased from an initial three sets at intensity 70% of 1-RM (6–11 repetitions per set) to 80% of 1-RM (6–8 repetitions per set) in the first seven weeks of the CR. In the LL-RE group, workload was increased from the initial 35% of 1-RM (12–22 repetitions per set) to 40% of 1-RM (12–16 repetitions per set). At exercise session 22 (after seventh week of training), we re-evaluated patients’ 1-RM in all three groups and the new maximal value was used to prescribe RE for the final five weeks of CR. Thus, the load in the HL-RE group progressed from 70% 1-RM (11 repetitions per set) to 80% 1-RM (6–8 repetitions per set), and the load in the LL-RE group progressed from 35% 1-RM (22 repetitions per set) to 40% 1-RM (12–16 repetitions per set) [21,26,27,28]. A lifting cadence of 1 s: 1 s (concentric and eccentric contraction) was used, with 90 s rest between sets [18]. Detailed progression of RE has been reported previously [21].

Patients were continuously monitored with beat-to-beat telemetry monitoring of heart rate and blood pressure before, during (throughout AE and after each set of RE) and after each training modality. All training sessions were supervised by a medical nurse and physiotherapist and guided by a kinesiologist, with a cardiologist available for consultations on site. Further details of the safety protocol and procedures of this study can be found elsewhere [21].

### 2.4. Measurements

#### 2.4.1. Monitoring of Exercise-Related Adverse Cardiovascular and Musculoskeletal Events

We closely monitored all potential exercise-related cardiovascular (dizziness, angina pectoris, blood pressure > 220/110 mmHg, palpitation, atrial fibrillation, arrhythmias, etc.) and musculoskeletal (muscle soreness and swelling; muscle, ligament, meniscus, tendon ruptures, tears and/or strains, and bone fractures) signs and symptoms that occurred during or after (<72 h) each measurement or training session. All major adverse events were evaluated by experienced consultant cardiologists and medical nurses for potential safety indications, which would require exclusion from this study. Patients were excluded from all activities in CR during the time of screening and were permitted to resume with training only after medical clearance [21].

#### 2.4.2. Adherence to the Training Intervention, Exercise Tolerance, Workload Data Collection and Analysis

During this study, we collected data on completed AE and RE sessions, while exercise tolerance (RPE) during RE was measured using the short version of Borgs’ scale (0–10) after each set [27]. Workload completed during AE was collected using SANA Sprint Plus software version 1.0.0 (Ergosana, Bitz, Germany). The software automatically collected the workload expressed in kilojoules (kJ = Watts × seconds × 10^3^). All training sessions were additionally manually checked and all unloaded intervals whereas patients were not cycling (e.g., quick rest room visit, short rest or during RT) were excluded from the final analysis. Workload completed during RE was also collected using spreadsheets that were in line with the prespecified progression of the training. The total workload of RE is expressed in kilograms [21]. In addition, we also noted adherence to the progression of AE and RE.

#### 2.4.3. Maximal Leg Press Strength Measurement

Leg press familiarization and submaximal strength tests were completed using a Life Fitness Leg Press Pro 2 (Life Fitness Inc., Rosemont, IL, USA) at baseline, following 7 weeks of training and post-training (Table 1). After a general warm-up (5 min cycling at 50% maximal heart rate with cadence 50–60 rpm and dynamic stretching of lower limbs), patients were shown correct lifting technique and were familiarized with the protocol for leg press testing. The test was performed with the patient in a seated position with their back in permanent contact with the seat back of the machine, with hands holding the handles of the machine, and hips and knee at 0° and 90° of flexion in the starting position. During the test, patients completed a warm-up set comprising eight and six repetitions at 50% and 70% of their perceived 1-RM, respectively. The weight was progressively increased until reaching the workload that could be lifted three to five times (3–5 RM), with a two–three min rest between the trials [28]. The 1-RM was calculated using the established 1-RM prediction equation (predicted 1-RM = maximal load lifted/1.0278 − 0.0278 × number of repetitions) [29].

#### 2.4.4. Heart Rate Response to Low-Load and High-Load Resistance Exercise

We performed measurement of acute HR response to LL-RE and HL-RE at baseline (before 2nd–3rd session) and post-training (before 34th and 35th session) in a crossover, randomized manner, which remained the same at post-training measurement (Table 1).

HR was measured using a Nellcor Oximax N-65 pulse oximeter (Covidien LLC, Manfield, MA, USA) at baseline (3 min before exercise), after each set and 3 min post-exercise; while RPE was reported using the a short version of Borgs’ scale (0–10) after each set [30].

Patients first completed a general warm-up and baseline measurement of resting HR followed by RE in line with the sequence of randomization. The exercises consisted of three sets of either 16 repetitions at 40% of 1-RM (LL-RE) or eight repetitions at 80% of 1-RM (HL-RE), with a lifting cadence ratio of 1 s of concentric contraction and 1 s of eccentric contraction, and with 90 s of rest between sets [17,18,28]. To eliminate the potential effects of the training load [28], we equated the cumulative load between LL-RE and HL-RE according to the maximal repetitions performed in HL-RE (eight repetitions at 80% of 1-RM) [19]. Patients performed the other type of RE following 48–72 h of rest. 

### 2.5. Statistical Analysis

Descriptive variables are presented as frequencies (%) and numeric variables are presented as the mean (standard deviation) or as the median (interquartile range), where appropriate. Assumptions of normality of distribution (Shapiro–Wilk test and histogram), homogeneity of variances (Levene’s test) and sphericity (Mauchly’s test) were checked for all numeric outcomes. In line with prespecified per-protocol analysis [21], we included all patients that completed at least 24 training sessions (e.g., 8 weeks) in the final analysis, as similar or longer (>12 weeks) duration of combined AE and RE was previously shown to be superior to AE alone in patients with CAD [5,6]. The difference between groups in training adherence was assessed using Fisher’s exact test. Between-group difference in average and cumulative workload during AE and RE was assessed using one-way analysis of variance (ANOVA). The effects of the training intervention on 1-RM, cumulative load at different RE intensities and hemodynamic adaptations to LL-RE and HL-RE were assessed using two- or three-way repeated-measures ANOVA (main outcomes: effects of time, group and/or load and effect of interactions), with additional between-group and within-group comparisons performed using Bonferroni correction for multiple comparisons. Statistical analysis was performed using IBM SPSS 25 software (SPSS Inc., Armonk, NY, USA) at a level of statistical significance set at alpha < 0.05.

## 3. Results

A total of 154 patients with CAD were screened for eligibility; of these, 79 were included in this study (Figure 1). For medical or personal reasons, 20 patients were not able to attend the rehabilitation sessions as planned, thus 59 patients were finally included in the analysis. The group were predominantly men (75%), 61 (8) years old, and had a left ventricular ejection fraction of 53 (9) %. Body mass index was higher in the AE group compared with the LL-RE group (+4.12 kg/m^2^; *p* = 0.010). Most patients were non-smokers or ex-smokers, with no between-group difference (*p* = 0.346). In the AE group, more patients were diagnosed with atrial fibrillation than in the HL-RE and LL-RE groups (*p* = 0.038). There was no other relevant between-group difference in baseline characteristics (Table 2). 

### 3.1. Safety of the Training Intervention

With the exception of a very few reports of short light headedness (4%) during baseline HL-RE and muscle soreness following baseline 1-RM and/or evaluation of hemodynamic response to LL-RE or HL-RE, there were no major cardiovascular events or complications (angina pectoris, blood pressure > 220/110 mmHg, palpitation, atrial fibrillation, arrhythmias) and no exercise-limiting musculoskeletal problems. In the AE group, two patients were unable to perform testing on leg press machine due to chronic lower back pain, one patient aggravated previous ischiatic pelvis pain and could not complete post-training 1-RM measurement and one patient failed to complete baseline evaluation of hemodynamic response to HL-RE and was excluded from the follow-up re-evaluation. All patients in the HL-RE and LL-RE groups had no exercise-related limitation at baseline and during the training intervention.

### 3.2. Adherence to Aerobic and Resistance Exercise Training

Apart from two patients in HL-RE with completed 24 visits, all other patients completed 36 visits to CR. Adherence to training was almost complete: eight patients failed to complete 36 AE sessions (AE group: one patient completed 35 sessions; LL-RE group: one patient completed 34 sessions and four patients completed 35 sessions; HL-RT: two patients completed 35 sessions), and only one patient failed to complete all HL-RE sessions (35 completed sessions). Adherence to AE protocol was good: only six patients failed to follow the progression of AE (AE group: four patients; LL-RE: five patients; HL-RE group: four patients), whereas adherence to RE was excellent (100%). Thus, there was no between-group difference in completed training sessions (*p* = 0.222) and adherence to AE (*p* = 0.106) and RE (*p* = 0.475) protocols.

### 3.3. Workload during Aerobic and Resistance Exercise Training

Patients on average completed 109 (357) kJ and lifted 3255 (2408, 3726) kg during each AE and RE session, respectively. In total, patients completed 3835 (1313) kJ during AE and lifted a total of 93,766 (66,713, 107,856) kg during RT. The average and cumulative workloads during AE and RE did not differ between training groups (Table 3).

### 3.4. Training Loading, Heart Rate and Rating of Perceived Exertion during Resistance Exercise

Cumulative RE workload, HR and RPE during different mesocycles of RE are presented in Table 3. Two-way ANOVA demonstrated a significant effect of time for cumulative workload (*p* < 0.001), HR change (*p* < 0.001) and RPE during RE (*p* = 0.001), and a significant effect of time x group interaction on RPE during RE (*p* < 0.001).

Despite no between-group difference in cumulative RE workload and increase in HR during the exercise, the RPE was significantly higher in the HL-RE group compared with the LL-RE group in the third (+1.1 points, *p* = 0.048), fourth (+1.9 points, *p* = 0.002), fifth (+1.9 points, *p* = 0.002) and sixth mesocycles (+2.0 points, *p* = 0.001) of RT (Table 4). The RE cumulative load significantly increased from the first to sixth mesocycles in LL-RE and HL-RE (all *p* < 0.01), with exception of no differences between the second and fifth mesocycles in the LL-RE group (*p* = 1.000).

RPE significantly decreased from the first to second (−0.5 points, *p* = 0.031) and third mesocycles (−0.9 points, *p* = 0.003), and additionally significantly decreased from the second to third mesocycles (−0.4 points, *p* = 0.045) in the LL-RE group. In the HL-RE group, RPE was significantly higher in the fourth to sixth mesocycles compared with the first (vs. fourth: +0.8 points, *p* = 0.024; vs. fifth: +0.9 points, *p* = 0.037), second (vs. fourth: +0.9 points, *p* < 0.001; vs. fifth: +1.0 points, *p* < 0.001; vs. sixth: +0.9 points, *p* = 0.010) and third mesocycles (vs. fourth: +1.1 points, *p* < 0.001; vs. fifth: +1.1 points, *p* < 0.001; vs. sixth: +1.0 points, *p* = 0.002), respectively.

Furthermore, the increase in HR during exercise was significantly greater in the fourth to sixth mesocycles compared with the first (vs. fourth: +6%, *p* = 0.037; vs. fifth: +7%, *p* < 0.001), second (vs. fifth: +6%, *p* = 0.005) and third mesocycles (vs. fourth: +7%, *p* = 0.003; vs. fifth: +8%, *p* < 0.001; vs. sixth: +4%, *p* = 0.048) in the LL-RE group. In the HL-RE group, the increase in HR during exercise was significantly greater in the fourth and fifth mesocycles compared with the first (vs. fourth: +6%, *p* = 0.039) and third mesocycles (vs. fourth: +9.0%, *p* < 0.001; vs. fifth: +6%, *p* = 0.001), respectively. 

### 3.5. Maximal Lower Limb Strength

**Figure 2:**
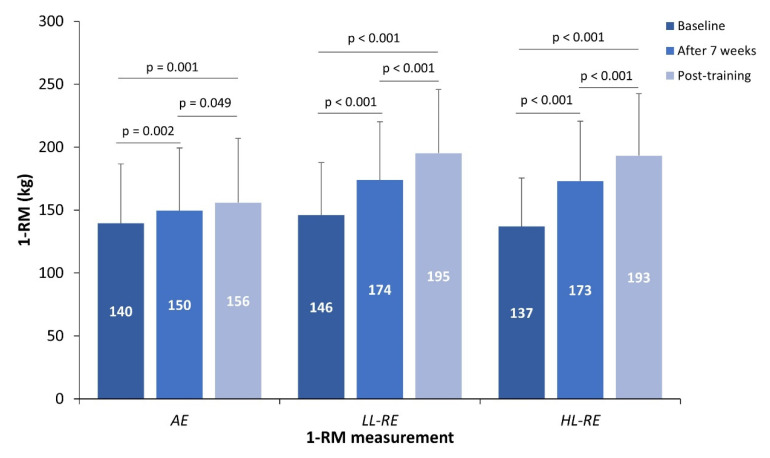
The 1-RM at baseline, after seven weeks and post-training.

The training intervention had a significant time effect (*p* < 0.001) and a time x interaction effect (*p* < 0.001) on 1-RM. Compared with baseline, all training groups significantly improved 1-RM after seven weeks and post-training (Figure 2). Improvement in 1-RM was significantly greater than in the HL-RE than in the LL-RE (+7%, *p* = 0.012) and AE groups (+20%, *p* < 0.001) after 7 weeks. In addition, 1-RM was also improved to a greater extent in the LL-RE group compared with the AE group (+13%, *p* < 0.001) after seven weeks. Following the training intervention, there was a significantly greater improvement in 1-RM in the HL-RE (+31%, *p* < 0.001) and LL-RE (+23%, *p* < 0.001) groups compared with the AE group.

### 3.6. Hemodynamic Response and Adaptations to Resistance Exercise

**Figure 3:**
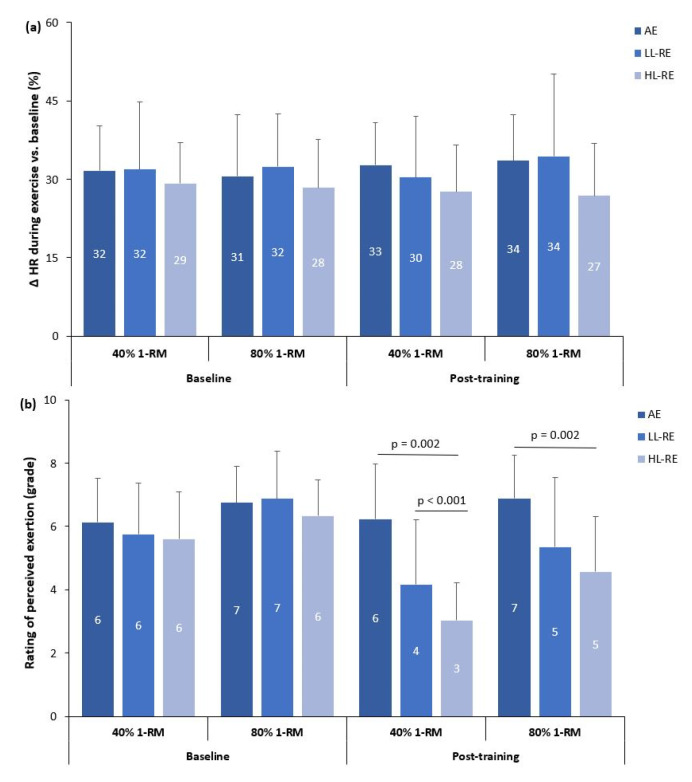
Heart rate (**a**) and exercise tolerance (**b**) adaptations to low-load and high-load resistance exercise. Δ HR: % change in heart rate during the resistance exercise; AE: aerobic exercise, LL-RE: low-load resistance training exercise; HL-RE: high-load resistance exercise.

Three-way ANOVA showed no significant effects of time (baseline, post-training) (*p* = 0.792), load (40% of 1-RM, 80% of 1-RM) (*p* = 0.575), group × load (*p* = 0.258), group × time (*p* = 0.271), load × time (*p* = 0.337) or time × group × load interaction (*p* = 0.726) on HR response during the RE (Figure 3a).

In contrast, there was a significant effect of time (*p* < 0.001; partial η^2^ = 0.450), load (*p* < 0.001; partial η^2^ = 0.611), and time × group (*p* < 0.001; partial η^2^ = 0.334), but non-significant effects of load × group (*p* = 0.122), time x load (*p* = 0.182) and time × group × load interaction (*p* = 0.282) on RPE during RE (Figure 3b).

There was no within-group, between-group or between-RE modality differences in HR response to RE during baseline and post-training measurements (Figure 3a).

At baseline, there was no significant difference between groups in RPE during LL-RE and HL-RE (Figure 3b). During baseline and post-training measurements, RPE was significantly higher during HL-RE compared with LL-RE in the AE (baseline: *p* = 0.044; post-training: *p* = 0.024), LL-RE (both *p* < 0.001) and HL-RE groups (baseline: *p* = 0.007; post-training: *p* < 0.001).

During post-training LL-RE, RPE was significantly higher in the AE group compared with the LL-RE and HL-RE groups (*p* < 0.01; Figure 3b). During post-training measurements of HL-RE, there was also a significantly higher RPE in the AE group compared with the HL-RE group (*p* = 0.002).

When compared with baseline measurements, RPE during post-training LL-RE and HL-RE was significantly lower compared in the LL-RE and HL-RE groups (all *p* < 0.001).

## 4. Discussion

Our study is the first to evaluate the safety, feasibility and efficacy of HL-RE and LL-RE when combined with AE and compared to AE alone in the early phase of CR of patients with CAD. HL-RE and LL-RE were both safe and well accepted among patients, although LL-RE was better tolerated. Adherence to both RE protocols was excellent, with no differences in cumulative RE volumes. Maximal muscle strength was improved to a greater extent in HL-RE compared with LL-RE and AE after 7 weeks of training, while such difference between the RE groups diminished following the training intervention. HL-RE and LL-RE improved exercise tolerance during post-training HL-RE and LL-RE measurement, but failed to elicit hemodynamic adaptations to RE. 

Despite poorly reported adverse events during AE and RE in CR [5,6,31], both training modalities were shown to be safe. Similar to the safety profile of AE established in our study, high-intensity AE was previously shown to have a very low rate of major adverse cardiovascular events (1 event/11,333 training hours) [31]. Additionally, none of the previous studied that applied combined high-intensity AE and RE reported any major cardiovascular events [5], which is also supported by our study. Most of the reported adverse events were minor, such as aggravations of previous chronic pain in lower back and knee. This was also observed in our study, whereas chronic lower back pain limited two patients to perform evaluation on leg press machine. While termination of RE due to chronic musculoskeletal issues was rare in previous studies [5,6], one patient in the AE group could not complete follow-up assessment of 1-RM on leg press machine.

Participation and adherence to CR has been associated with reduced myocardial reinfarction rate, cardiovascular and all-cause mortality [32,33]. Despite such importance, participation in CR is often limited by the lack of transportation to the rehabilitation center [34], which was also one of the main limiting factors for inclusion to our study (31% of the excluded patients). During this study, 20 patients were lost to follow-up, thus, the drop-out rate was greater (25%) than previously reported across European CR centers (15%) [35]. The drop-off rate in our study was largely impacted by the discontinuation of CR due to first national coronavirus-19 lockdown, thus, this may explain the discrepancy compared with previous findings [35].

In our study, the adherence to AE was high and to RE protocols was excellent. During this study, only 28 out of 2091 AE sessions (1.3%) were not completed according to the AE protocol. Patients that failed to comply with AE protocol were older with multiple chronic cardiovascular and musculoskeletal comorbidities. The same patients’ characteristics were also previously demonstrated to be a limiting for participation and adherence to exercise-based CR [34]. In addition, most of the AE sessions that were not performed in compliance with the protocol were performed during the last part of this study, wherein AE intensity was high (>74% of peak power output) [21]. Therefore, it seems that the intensities close to high-intensity interval AE may be associated with lower protocol adherence, especially since the moderate-intensity interval AE was shown to be more tolerable [36]. Nevertheless, the adherence to our progressive moderate- to high-intensity AE was still much higher than reported previously in patients with cardiovascular diseases [36], which further promotes the feasibility of our AE program. Furthermore, adherence to RE protocols was excellent and similar as in most of the previous studies that compared HL-RE and LL-RE in healthy older adults [14]. Additionally, our study also demonstrated similar adherence to RE as previously demonstrated following combined RE and AE in patients with CAD [27].

Majority of the previous studies in healthy older adults that evaluated the dose-dependent response between RE load and improvements in muscle strength balanced the cumulative training volume by manipulating other training variables (e.g., number of sets and repetitions per set); however, most of them failed to report the exact training volumes [14]. Nevertheless, two of those studies showed similar cumulative training workload achieved in LL-RE and HL-RE in healthy older adults [37,38]. With the lack of evidence on training workloads in patients with CAD, our study was the first to replicate findings established in healthy peers. Throughout this study, the completed RE workload remained similar between the RE groups and increased in line with the progression of RT.

The implementation of RE in CR, regardless of intensity, was shown to be associated with improvement in maximal muscle strength in patients with CAD [5,6]. Since the previous studies in CR mostly applied low to moderate loads in RT [5,6], our study was one of a few that applied HL-RT [27,39,40] and showed comparable improvement in 1-RM (LL-RT: +36%; HL-RT: 43%) as demonstrated in previous progressive moderate- to HL-RE intervention (+25–43%) in patients with CAD [27,40]. In addition, the combined training interventions were more effective in women undergoing longer training interventions (>6 months) [40] and in frail patients following coronary artery bypass surgery [39] than in men with stable CAD following stenting [27], with similar clinical characteristics to the patients in our study.

Previous meta-analysis has shown the dose-dependent relationship between RE load and improvement in maximal muscle strength can be attenuated by the variation in cumulative training volume of LL-RE and HL-RE [14]. Therefore, most previous randomized studies in healthy older adults equated the training volumes between HL-RE and LL-RE and showed no between-group difference in post-training improvement in maximal muscle strength [14]. The same methodological approach to the prescription of RE was also applied in our study, wherein we have to some extent replicated the previous findings with our post-training results. In contrast, we have demonstrated a greater improvement in 1-RM in HL-RE compared with LL-RE following the first 7 weeks of the intervention. Similarly, we have also demonstrated a greater improvement in maximal voluntary contraction of knee extensors (primary outcome of our study) following HL-RE compared with LL-RE or AE alone [41]. Such discrepancies can be linked to the method of maximal muscle strength measurement used across studies (i.e., 3–5 RM prediction of maximal strength versus maximal testing of muscle strength/torque) [14] and a possible motor learning effect associated with cortical reorganization [42], as a result of multiple repetitions/sessions performed on the same training device (e.g., leg press machine and leg curl).

Moderate- to HL-RE has been recommended only recently in CR [1,2], despite available evidence demonstrating its safety with lower hemodynamic response compared with traditionally advised LL-RE [16,17] in patients with CAD. While all hemodynamic studies were performed in CAD patients with previous training experiences within CR [16,17,18], we have recently demonstrated that similar hemodynamic response to HL-RE and LL-RE occurred also in patients with CAD prior to enrolment to CR [19]. Since previous studies focused mostly on the effects of RE on resting HR and blood pressure [43,44,45], only two previous studies have evaluated the hemodynamic adaptations during RE following combined AE and RE [45,46]. In line with our findings, both studies showed similar HR response to both RE at baseline and following combined exercise training [45,46].

RPE is a subjective measure for rating of exercise intensity [30] and was shown to be highly associated with RE load [47]; therefore, its use is also advised in CR settings for (frail) patients with CAD [1]. However, only a few studies are available in healthy adults, elderly and CAD patients and have shown conflicting results [16,48,49]. In line with findings in healthy older adults [48], we have demonstrated similar RPE between baseline HL-RE and LL-RE, while other studies have shown a greater RPE during HL-RE compared with LL-RE [16,49]. The discrepancies in findings between studies can be explained with the differences in age, training status of the participants and training intensities applied in LL-RE and HL-RE. Furthermore, participation in HL-RE and LL-RE improved exercise tolerance during post-training HL-RE and LL-RE, which was also previously established following combined AE and progressive moderate-to HL-RE in patients with CAD [45]. 

Some limitation of this study must be acknowledged. The feasibility outcomes of this study were limited by subjectively assessment of exertion and monitoring of cumulative training volume of AE and RE. This study was powered only for comparisons between the combination of AE and HL-RE or LL-RE and AE alone; therefore, all comparisons between the RE groups are only exploratory. To ensure patients’ safety, our 1-RM measurements were limited to only prediction of maximal leg press strength values (e.g., 3–5 RM testing). Moreover, the evaluation of hemodynamic response to HL-RE and LL-RE was limited to only evaluation of HR using pulse oximetry, while blood pressure adaptations should be in future studies evaluated using photoplethysmography. In addition, hemodynamic adaptations were not controlled for the change in potential beta blocker therapy following the intervention; however, similar confounders were also not controlled in previous hemodynamic studies of patients with CAD [16,17,18,19]. Finally, the ambulatory safety precautions during the coronavirus-19 restriction (e.g., mandatory face mask wear) may have impacted the results of exercise tolerance and hemodynamic response to RE.

## 5. Conclusions

In conclusion, the addition of HL-RE and LL-RE to AE was shown to be safe, well tolerated and associated with a similar improvement in the predicted maximal muscle strength of lower limbs, and hemodynamic response and adaptations within the physiological range. LL-RE was better tolerated than HL-RE; therefore, LL-RE seems to be more suitable for frail and/or sarcopenic patients with CAD to build baseline muscle conditioning prior to progression to higher intensities of RE, while HL-RE can be applied for well-conditioned middle-aged patients with CAD. Exercise tolerance was considerably improved following LL-RE and HL-RT, which further supports the addition of RE programs to standard exercise-based CR for patients with CAD. Further research is needed to establish new evidence on safety of early implementation of HL-RE in CR and its role on hemodynamic adaptations following training in patients with various cardiovascular diseases.

## Figures and Tables

**Table 1:** Study measurements and training protocol overview.

Study Visit	Measurement or Training Session
1. visit	Diagnostic screening (7–14 days prior to enrolment)
2. visit	Familiarization with leg press exercise (3–10 days prior to enrolment)
1. training session	1-RM measurement followed by AE: AE: interval cycling (5 min workload/2 min active recovery) at 50% of peak power output
2.–3. training session	Haemodynamic response to HL-RE (80% of 1-RM) and LL-RE (40% of 1-RM) followed by AE: interval cycling (5 min workload/2 min active recovery) at 50% of peak power output
4.–11. training session (**1. mesocycle**)	HL-RE: 3 sets, 6–11 reps/sets, 70% of 1-RM LL-RE: 3 sets, 12–22 reps/sets, 35% of 1-RM AE: interval cycling (5 min workload/2 min active recovery) at 50–56% of peak power output
12.–16. training session (**2. mesocycle**)	HL-RE: 3 sets, 8–10 reps/sets, 75% of 1-RM LL-RE: 3 sets, 16–20 reps/sets, 37.5% of 1-RM AE: interval cycling (5 min workload/2 min active recovery) at 58–62% of peak power output
17.–20. training session (**3. mesocycle**)	HL-RE: 3 sets, 6–8 reps/sets, 80% of 1-RM LL-RE: 3 sets, 12–16 reps/sets, 40% of 1-RM AE: interval cycling (4–5 min workload/2 min active recovery) at 62–68% of peak power output
22. training session	1-RM measurement followed by AE: interval cycling (4 min workload/2 min active recovery) at 68% of peak power output
23.–24. training session (**4. mesocycle**)	HL-RE: 3 sets, 11 reps/sets, 70% of 1-RM LL-RE: 3 sets, 22 reps/sets, 35% of 1-RM AE: interval cycling (4 min workload/2 min active recovery) at 68–70% of peak power output
25.–28. training session (**5. mesocycle**)	HL-RE: 3 sets, 9–10 reps/sets, 75% of 1-RM LL-RE: 3 sets, 18–20 reps/sets, 37.5% of 1-RM AE: interval cycling (4 min workload/2 min active recovery) at 72–74% of peak power output
29.–33. training session (**6. mesocycle**)	HL-RE: 3 sets, 6–8 reps/sets, 80% of 1-RM LL-RE: 3 sets, 12–16 reps/sets, 40% of 1-RM AE: interval cycling (3–4 min workload/2 min active recovery) at 74–80% of peak power output
34.–35. training session	Haemodynamic response to HL-RE (80% of 1-RM) and LL-RE (40% of 1-RM) followed by AE: AE: interval cycling (3 min workload/2 min active recovery) at 80% of peak power output
36. training session	1-RM measurement followed by AE: AE: interval cycling (3 min workload/2 min active recovery) at 80% of peak power output

AE: aerobic exercise; LL-RE: low-load resistance exercise; HL-RE: high-load resistance exercise; 1-RM: one-repetition maximum.

**Table 2:** Baseline sample demographic, anthropometrical and clinical characteristics.

Variable	Sample (*n* = 59)	AE Group (*n* = 19)	LL-RE Group (*n* = 19)	HL-RE Group (*n* = 21)	*p* (ANOVA)
Sex (females, (%))	14 (25)	5 (16)	4 (21)	6 (29)	0.931
Age (years)	61 (8)	61 (9)	61 (7)	62 (8)	0.910
Anthropometrics					
Height (cm)	172.1 (8.4)	170.4 (8.8)	172.8 (8.6)	172.9 (7.9)	0.582
Weight (kg)	85.47 (15.43)	90.94 (19.04)	81.46 (13.37)	84.15 (12.56)	0.148
Body mass index (kg/m^2^)	28.81 (4.47)	31.25 (5.71)	27.13 (3.04)	28.81 (3.39)	0.010
Clinical data					
LVEF (%)	53 (9)	50 (45, 60)	55 (50, 60)	50 (45, 58)	0.454
Time from clinical event to inclusion to CR (months)	2.0 (1.5, 3.0)	2.0 (2.0, 2.5)	2.5 (1.5, 3.0)	2.0 (1.5, 2.8)	0.832
Myocardial infarction, f (%)					
NSTEMI	25 (42.37)	9 (47.4)	8 (42.1)	8 (38.1)	0.947
STEMI	24 (40.68)	7 (36.8)	7 (36.8)	10 (47.6)	
Unstable AP/PCI	10 (16.95)	3 (15.8)	4 (21.1)	3 (14.3)	
Comorbidities and risk factors, f (%)					
Arterial hypertension	41 (69.49)	15 (78.9)	11 (57.9)	15 (71.4)	0.383
Hyperlipidemia	49 (83.10)	16 (84.2)	14 (73.7)	19 (90.5)	0.384
Diabetes	9 (15.25)	4 (21.1)	3 (15.8)	2 (9.5)	0.602
Atrial fibrillation	5 (8.48)	4 (21.1)	1 (5.3)	0 (0.0)	0.038
Thyroid disease	5 (8.48)	2 (10.5)	2 (10.5)	1 (4.8)	0.727
Renal disease	4 (6.78)	0 (0.0)	2 (10.5)	2 (9.5)	0.534
Smoking, f (%)					
Non-smoker	14 (23.73)	3 (15.8)	3 (15.8)	8 (38.1)	0.346
Ex-smoker	35 (59.32)	13 (68.4)	11 (57.9)	11 (52.4)	
Smoker	10 (16.95)	3 (15.8)	5 (26.3)	2 (9.5)	
Pharmacological therapy, f (%)					
Aspirin	57 (96.60)	17 (89.5)	19 (100.0)	21 (100.0)	0.200
Beta blocker	59 (100.00)	19 (100.0)	19 (100.0)	21 (100.0)	1.000
ACE inhibitor/ARB	58 (98.30)	19 (100.0)	18 (94.7)	21 (100.0)	0.644
Statin	59 (100.00)	19 (100.0)	19 (100.0)	21 (100.0)	1.000
Antiplatelet drug	58 (98.30)	18 (94.7)	19 (100.0)	21 (100.0)	0.644
Anticoagulation drug	5 (8.48)	3 (15.8)	1 (5.3)	1 (4.8)	0.509
Diuretic	5 (8.48)	4 (21.1)	0 (0.0)	1 (4.8)	0.071

Data are presented as the mean (standard deviation) or as the median (first quartile, third quartile). AE: aerobic exercise; LL-RE: low-load resistance exercise; HL-RE: high-load resistance exercise; LVEF: left ventricular ejection fraction; (N)STEMI: (non)ST-segment-elevated myocardial infarction: AP: angina pectoris; PCI: percutaneous coronary intervention; ASA: acetylsalicylic acid; ACE: angiotensin-converting enzyme; ARB: angiotensin II receptor blockers.

**Table 3:** Mean and cumulative workload during aerobic and resistance training.

Variable	Group	Mean (SD)	Test Statistics
Mean AE workload (kJ)	AE	117 (37)	F = 1.658 *p* = 0.200
	LL-RE	113 (35)	
	HL-RE	98 (34)	
Cumulative AE workload (kJ)	AE	4175 (1352)	F = 2.056 *p* = 0.138
	LL-RE	3987 (1242)	
	HL-RE	3388 (1274)	
Mean RE workload (kg)	LL-RE	3091 (856)	t = 0.703 *p* = 0.487
	HL-RE	3016 (797)	
Cumulative RE workload (kg)	LL-RE	89,505 (24,949)	t = 0.285 *p* = 0.777
	HL-RE	84,092 (23,765)	

AE: aerobic exercise, LL-RE: low-load resistance exercise; HL-RE: high-load resistance exercise; kJ-kilo Joule.

**Table 4:** Cumulative workload, heart rate and rating of perceived exertion during each mesocycle of resistance exercise training.

Variable	Group	1. Part of This Study (Mesocycles)			2. Part of This Study (Mesocycles)			Time Effect	Time × Group
		1.	2.	3.	4.	5.	6.		
Cumulative RE workload (kg)	LL-RE (*n* =19)	22,372 (6472)	15,514 (4410)	12,991 (3711)	8014 (2176)	15,221 (4152)	15,394 (4201)	*p* < 0.001 η^2^ = 0.915	*p* = 0.188 η^2^ = 0.047
	HL-RE (*n* = 19)	21,013 (5871)	14,494 (4048)	12,187 (3423)	8022 (2161)	15,207 (4171)	15,391 (4149)		
Δ HR during RE vs. pre-exercise (%)	LL-RE (*n* =19)	29 (10)	30 (13)	28 (13)	35 (15)	36 (14)	32 (13)	*p* < 0.001 η^2^ = 0.322	*p* = 0.555 η^2^ = 0.020
	HL-RE (*n* = 19)	26 (8)	26 (9)	23 (8)	32 (12)	29 (11)	27 (9)		
RPE during RE (point)	LL-RE (*n* =19)	5 (2)	5 (2)	4 (2)	5 (2)	5 (2)	4 (2)	*p* = 0.001 η^2^ = 0.172	*p* < 0.001 η^2^ = 0.214
	HL-RE (*n* = 19)	6 (1)	5 (1)	5 (1)	6 (1)	6 (1)	6 (1)		

Data are presented as the mean (standard deviation). LL-RE: low-load resistance exercise; HL-RE: high-load resistance exercise; 1-RM: one-repetition maximum; Δ HR: % change in heart rate (Δ HR = HR during exercise-HR pre-exercise/HR pre-exercise × 100%); η^2^: partial eta squared.

## Data Availability

The supporting data for this study are available from the corresponding author upon reasonable request.
